# Etiology and pattern of zygomatic bone fracture among patients with facial injuries attending Muhimbili National Hospital, Tanzania

**DOI:** 10.11604/pamj.2025.52.13.42463

**Published:** 2025-09-11

**Authors:** Paulo Joseph Laizer, David Kiwango Deoglas, Sira Owibingire

**Affiliations:** 1 Department of Oral and Maxillofacial Surgery, School of Dentistry, Muhimbili University of Health and Allied Sciences, P.O. Box 65001, Dar es Salaam, Tanzania

**Keywords:** Zygomatic bone fracture, fracture pattern, fracture management

## Abstract

The zygomatic bone is frequently fractured following maxillofacial injuries due to its prominence. Fracture of this bone has functional, cosmetic, and psychological effects. Proper management is therefore crucial. Data on the etiology and pattern of zygoma fracture will aid in planning management and setting appropriate preventive measures. The study aimed to determine the etiology and pattern of zygomatic bone fractures among patients with facial injuries attending Muhimbili National Hospital. We carried out a prospective hospital-based cross-sectional study where a consecutive sampling of patients with facial injuries attending Muhimbili National Hospital from May 2021 to March 2022 was done. We interviewed patients with facial injuries on their socio-demographics and etiology of injury using a pre-tested self-administered questionnaire. Thereafter, we used a CT scan of the head to record the pattern of the zygomatic bone fracture. Data were entered in the SPSS computer program version 20.0 for analysis and were presented in the form of frequency distribution tables, graphs, and means. We examined 420 patients with facial injuries; among them, 137 patients had zygomatic bone fractures. The majority were males, and the male-to-female ratio was 7.5:1. Most fractures were found in the age group 20-29 years. The majority of fractures were due to road traffic accidents (RTA). Most patients had no significantly displaced fractures, and fractures with orbital floor involvement were rare. The maxillary bone was the most common concomitant fracture. Zygomatic bone fractures occurred mostly in males in their third decade. Road traffic accidents account for most fractures. Most fractures had no significant displacement. Fractures with orbital floor disruption were rare. Maxillary bone was a common concomitant fracture.

## Introduction

The zygomatic bone forms the prominences of the cheek, orbital wall, and the buttresses of the face. It aids in the passage to neurovascular structures and provides structural support to muscles and fascia. Fracture of this bone is associated with visual impairment, facial disfigurement, trismus, masticatory dysfunction, and paresthesia [1]. Globally, zygomatic bone fracture is the second common facial bone fracture, just after the nasal bone. In Africa, the prevalence ranges from 25.6% to 34.7% [2]. Unlike developed countries, where the occurrence involves a wide range of age groups and occupations, in most developing countries, fracture of the zygomatic bone occurs mostly among young males who are motorcyclists. Most of the fractures occur among city dwellers with a low level of education and income. In Tanzania, zygomatic bone fractures constitute 26.8% of midfacial fractures [3].

There is a marked increase in the incidence of trauma involving the facial region in most of the developing African countries [3]. Road traffic accidents have been implicated in most cases; however, other etiologies haven´t been well explored. Studies revealed that etiologies of zygoma fractures vary according to the level of development of the country. Assault and sport-related accidents were found to be the common etiologies in most of the developed countries [4]. On the other hand, fractures as a result of road traffic accidents and domestic violence were seen mostly in low- and middle-income countries [5]. However, currently, there is a massive road and railway infrastructure development in most of these countries. These developments could have significantly improved road safety and therefore decreased the incidence of road accidents. The etiological data and trends of facial injuries should be updated to match the current environmental and socioeconomic changes in most African countries. Knowledge of the etiologies of zygoma fractures is important mainly in setting measures that are suitable for the prevention of facial injuries in a particular region/country.

Most of the zygomatic bone fractures are complex fractures involving its sutures; isolated fractures of the zygoma arch are rare [1]. The body of the zygoma is thick and highly compact, a major buttress of the face; as a result, fractures involving the body are extremely rare [3]. Most zygoma fractures are associated with the fracture of other facial bones. Maxillary bone fracture was the most frequent concomitant fracture [6]. The pattern of fracture of the zygoma depends on the magnitude and direction of the force directed to the midface. The etiological shift might be associated with changes in the pattern of fractures. The pattern of the fractures guides a surgeon to the management of the fracture and the anticipated complications. The aim of this study is therefore to find out the etiologies and patterns of zygomatic bone fractures in Tanzania. These findings are crucial in setting preventive measures suitable for this region and as guidance for surgical management.

## Methods

**Study setting:** this study was carried out at the Oral Maxillofacial Department of the Muhimbili National Hospital (MNH). The oral and maxillofacial surgery department has one main operating theatre with five operating days per week. The department is the main referral centre in the country with more than 15 specialists. On average, one patient with maxillofacial trauma is operated on per day.

**Study design:** this was a cross-sectional descriptive hospital-based study.

**Study duration:** this study was carried out from May 2021 to March 2022.

**Study population:** all patients with facial injuries who attended MNH between May 2021 and March 2022, who met the inclusion criteria, and consented to participate in this study.

**Inclusion criteria:** all patients with facial injuries attended the MUHAS Dental Clinic within 7 days of their injury.

**Exclusion criteria:** patients with facial injuries who, due to the extent of the injury, were incapable of providing information, as they needed immediate orthopedic, neurological, and intensive care unit management.

**Sampling procedure:** consecutive sampling of patients with facial injuries who attended MUHAS Dental Clinic, who met the inclusion criteria and consented to participate in this study, was done.

**Data collection:** the principal investigator and a research assistant participated in the identification of patients with facial injuries at MUHAS Dental Clinic. Patients were approached, and informed consent was sought. Patients were interviewed on their socio-demographic data, etiology, type of vehicle involved in an accident, site where injury occurred, and time of injury. The diagnosis of zygomatic bone fracture was confirmed using a CT scan. Interpretation of these radiographs was done by the principal investigator under supervision of an oral and maxillofacial radiologist. The pattern of fracture was classified using the Rowe and Killey classification, which is a widely accepted classification system for the fracture of the zygoma.

### Rowe and Killey classification for zygomatic bone fracture

**Class I:** fracture with no significant displacement.

**Class II:** isolated fracture of zygomatic arch.

**Class III:** fracture with rotation along vertical axis, i.e. inward or outward displacement of the orbital rim.

**Class IV:** fracture with rotation along the longitudinal axis, i.e. medial or lateral displacement of the frontal process.

**Class V:** en bloc displacement of the zygoma bone.

**Class VI:** fractures with orbital floor disruption/displacement.

**Class VII:** fractures with displacement of the orbital rim.

**Class VIII:** fracture with complex comminution of the zygoma bone.

**Data analysis:** the collected data were checked for their completeness, entered, edited, cleaned, and analyzed by statistical software SPSS Version 20. Data was presented in the form of frequency tables, graphs, and means. Association between variables was determined using the test. The probability (p) value of <0.05 was considered statistically significant.

**Variables:** i) social demographic characteristic (i.e. age, sex, locality, education level, marital status, and occupation); ii) etiology of zygomatic bone fracture (i.e. RTA, assault, animal attack, sport-related accidents, occupational-related accidents, domestic violence, and accidental fall); iii) pattern of fracture (i.e. bilateral, unilateral, left side, right side, concomitant fractures, Rowe and Killey classes).

**Ethical consideration:** ethical clearance (MUHAS-REC-06-2021-724) was obtained from the Muhimbili University of Health and Allied Sciences Senate Research and Publication Committee. All eligible patients presenting were enrolled after obtaining signed informed consent from them. Numbers were used for identification instead of patients´ names.

## Results

During this study period, we examined 420 patients with facial injuries; among them, 137 patients had zygomatic bone fractures. The majority, 121 (88.3%) of patients with zygomatic bone fractures were males, and the male-to-female ratio was 7.5:1. The age ranged from 8 to 64 years and the mean age was 31.35±10 years. Most fractures, 66 (48.2%), were found in the age group 20-29 years. Few patients were younger than twenty and older than sixty years (Table 1).

**Table 1:** distribution of patients with zygomatic bone fractures according to their socio-demographic characteristics

Age groups (years)	Gender of the patients		No. of patients (%)
	Male	Female	
<20	5	0	5 (3.6%)
20-29	58	8	66 (48.2%)
30-39	33	5	38 (27.7%)
40-49	15	2	17 (12.4%)
50-59	7	1	8 (5.8%)
60+	3	0	3 (2.2%)
**TOTAL**	121 (88.3%)	16 (11.7%)	137 (100%)
**Socio-demographic characteristics**		**Frequency**	**Percentage (%)**
**Locality**	Urban	104	75.9
	Rural	33	24.1
**Education level**	No formal education	6	4.4
	Primary education	73	53.3
	Secondary education	38	27.7
	Tertiary education	20	14.6
**Marital status**	Single	61	44.5
	Married	69	50.4
	Widow/widower	0	0
	Divorced	2	1.5
	Cohabiting	5	3.6
**Occupation**	No formal employment	12	8.8
	Civil servant	13	9.5
	Businessperson	21	15.3
	Self employed	46	33.6
	Private employee	21	15.3
	Student	9	6.6
	Peasant	15	10.9

**Etiology and associated factors:** the majority of fractures, 115 (83.9%), were caused by road traffic crashes; assault was the second most common etiology. Fractures as a result of accidental falls, occupational-related, and sport-related accidents were few (Table 2). Motorcycles were the commonly involved vehicle, 87 (63.5%). Motorcyclists who sustained injuries that led to zygoma fractures were 56 (40.9%), and motorcycle passengers, 40 (29.2%). Zygoma fractures due to car accidents were few. Most injuries 92, 67.1%) occurred during the evening and at night. Few injuries occurred during the afternoon (Table 2).

**Table 2:** distribution of patients with zygomatic bone fracture according to the cause and time of injury

Cause of injury	Time of injury				Total
	Morning	Afternoon	Evening	Night	
RTA	30 (26%)	9 (7.8%)	41 (35.6%)	35 (30.4%)	115 (83.9%)
Assault	2 (11.7%)	1 (5.9%)	5 (29.4%)	9 (52.9%)	17 (12.4%)
Sport-related accidents	0 (0.0%)	0 (0.0%)	1 (100%)	0 (0.0%)	1 (0.7%)
Occupationally related accidents	1 (50%)	0 (0.0%)	1 (50%)	0 (0.0%)	2 (1.4%)
Accidental fall	1 (50%)	1 (50%)	0 (0.0%)	0 (0.0%)	2 (1.4%)
**Total**	34 (24.8%)	11 (8%)	48 (35%)	44 (32.1%)	137 (100%)

RTA: road traffic accidents

**Figure 1:**
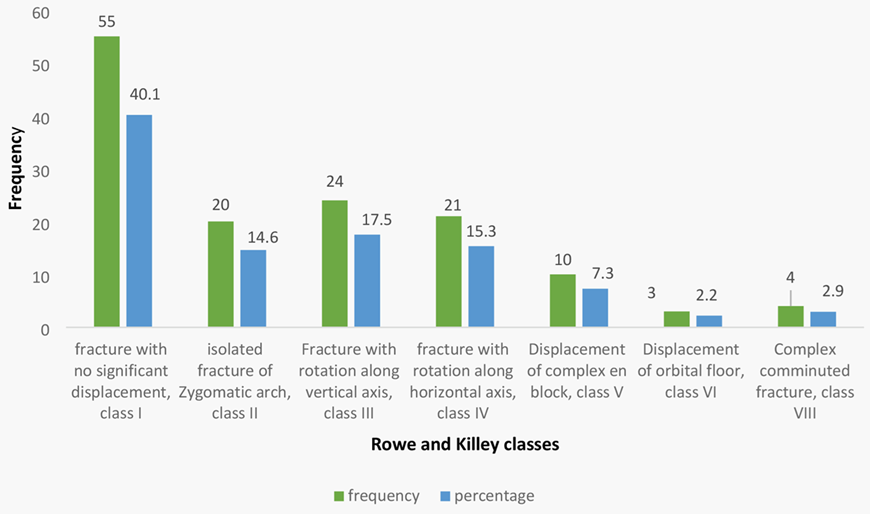
distribution of patients according to types of fracture management and Rowe and Killey’s classes

**Pattern of fracture:** the majority, 109 (79.5%) of fractures were unilateral, and there was a marked left-sided predominance, 65 (47.4%). Few fractures, 28 (20.4%), occurred bilaterally. Most fractures were in Rowe and Killey´s classes one and two (Figure 1). Isolated fractures of the zygomatic arch were found in 20 (14.6%). Fractures involving the orbital floor displacement were few (Figure 1).

**Figure 2:**
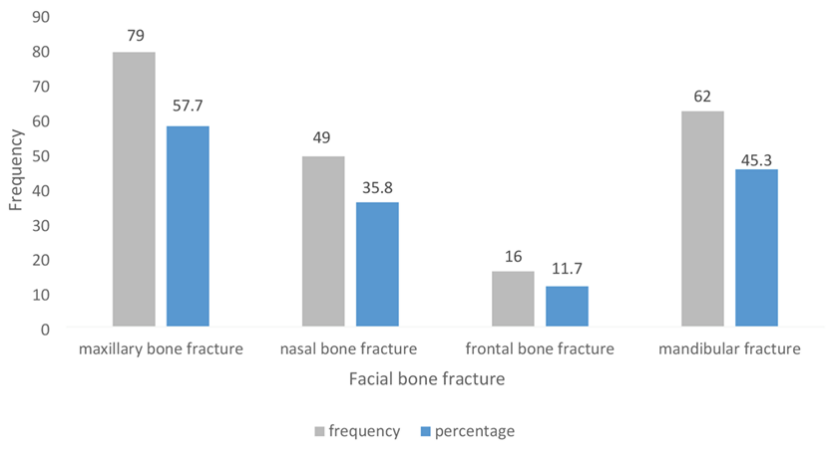
frequency of facial bones that fractured concomitantly with the zygomatic bone

**Concomitant fractures:** the maxilla was also found to fracture in 79 (57.7%) patients with zygomatic bone fracture (fig. 02). Mandibular fractures were found in 62 (45.3%), and nasal bone in 49 (35.8%). The frontal bone was the least likely bone to fracture concomitantly with the zygomatic bone (Figure 2). Displaced zygoma fractures were most likely to present with concomitant fractures (p=0.006).

## Discussion

The finding that zygomatic bone fracture is prevalent among males in their 3^rd^ and 4^th^ decades correlates with the study by Zaleckas *et al*. (2015) [7]; however, one study found a relatively low sexual predilection [8]. The high proportion of males with zygomatic bone fractures in this study could be explained by the fact that most of the motorcyclists were males, and motorcycles were the most involved vehicle in MTC. In addition, males are often involved in reckless and aggressive behaviours such as dangerous sports, violence, and reckless driving. Few patients were younger than 18 years or older than 49 years (Table 1). Similarly, one study reported similar findings [9]. Children are not allowed to ride or drive, and old people are more careful and less likely to be involved in aggressive behaviours, which could predispose them to accidents and consequently injuries.

In our study, 115 (83.9%) of zygomatic bone fractures were due to RTA. Similar findings have been reported in a study among Tanzanian patients as well as in most developing countries [3]. To the contrary, assault was found to be the commonest cause of zygomatic bone fracture in developed countries. In this study, motorcycles contributed to 63.5% of all road traffic crashes. This finding is consistent with several other studies [3,8]. Most 126 (92%) patients with zygomatic bone fractures in our study got injured during the evening, at night, and in the morning, which corresponds to the times for routine movements of people from their homeplaces to work and vice versa; a few 11 (8%) injuries occurred in the afternoon. A similar finding was reported by Zaleckas *et al*. (2015) [7]. In our study, most of the zygomatic bone fractures were unilateral with a left-sided bias, and only a few patients presented with bilateral involvement. The left-sided bias was also reported in Lithuania [7].

The majority of the zygomatic bone fractures in our study, 62 (45.3%), were displaced, while those with no significant displacement were 40.1%. Similar findings have been reported in one [5,10]. Isolated fractures of the zygomatic arch were found in a few (14.6%) patients, as similarly indicated in another study [1]. Displaced zygoma fractures are frequently found in high-impact trauma, as in this case, RTC. In our study, maxillary bone was found to fracture concomitantly with the zygomatic bone in most patients (79, 57.7%). In contrast, a study by Obuekwe *et al*. (2005) reported that the mandible was the bone that commonly got fractured simultaneously with the zygoma [8].

### Recommendation

A 3D CT scan is useful in the diagnosis and classification of zygomatic bone fractures. Further research on why motorcycles are commonly involved in motor traffic crashes that result in facial injuries should be carried out. The authorization of motorcycle use for public transport should be reviewed.

### Study limitation

Some of the patients couldn´t afford to do a CT scan due to financial reasons and therefore, were dropped from the study. Although the Muhimbili is a national referral hospital, the study being done at one health facility may not give a general picture of the zygoma bone fracture pattern in Tanzania. A broader study may be required to fulfil this limit.

## Conclusion

Zygomatic bone fractures occurred mostly in males in their third decade of life. Road traffic accidents, particularly motorcycle crashes, accounted for most of the fractures. Left side fractures were predominant. Most fractures were displaced, and isolated fractures of the zygomatic arch were few. Fractures with orbital floor disruption were rare. Maxillary bone was a common concomitant fracture.

### What is known about this topic

- Road traffic accident, assault, sport-related accident, and domestic violence are the common etiologies of zygoma fractures and right-sided predominance;
- Fractures were without significant displacement.

### What this study adds

- Zygoma fractures concomitantly occur with maxillary bone fractures;
- Fractures with orbital floor displacement are rarely found in patients with zygoma bone fractures;
- Despite the improvement in road infrastructures, road traffic accidents, particularly motorcycle accidents, remain the main cause of facial injuries that result to zygoma bone fractures.
